# VOCs Sensing by Metal Oxides, Conductive Polymers, and Carbon-Based Materials

**DOI:** 10.3390/nano11020552

**Published:** 2021-02-22

**Authors:** Milena Tomić, Milena Šetka, Lukaš Vojkůvka, Stella Vallejos

**Affiliations:** 1 Institute of Microelectronics of Barcelona (IMB-CNM, CSIC), Campus UAB, 08193 Cerdanyola del Vallès, Barcelona, Spain; milena.tomic@imb-cnm.csic.es; 2 Department of Electronic Engineering, Autonomous University of Barcelona (UAB), Campus UAB, 08193 Cerdanyola del Vallès, Barcelona, Spain; 3 CEITEC—Central European Institute of Technology, Brno University of Technology, 61200 Brno, Czech Republic; milena.setka@ceitec.vutbr.cz; 4 Silicon Austria Labs, Microsystem Technologies, High Tech Campus Villach, Europastraβe 12, A-9524 Villach, Austria; Lukas.Vojkuvka@silicon-austria.com

**Keywords:** volatile organic compounds, gas sensors, nanomaterials

## Abstract

This review summarizes the recent research efforts and developments in nanomaterials for sensing volatile organic compounds (VOCs). The discussion focuses on key materials such as metal oxides (e.g., ZnO, SnO_2_, TiO_2_ WO_3_), conductive polymers (e.g., polypyrrole, polythiophene, poly(3,4-ethylenedioxythiophene)), and carbon-based materials (e.g., graphene, graphene oxide, carbon nanotubes), and their mutual combination due to their representativeness in VOCs sensing. Moreover, it delves into the main characteristics and tuning of these materials to achieve enhanced functionality (sensitivity, selectivity, speed of response, and stability). The usual synthesis methods and their advantages towards their integration with microsystems for practical applications are also remarked on. The literature survey shows the most successful systems include structured morphologies, particularly hierarchical structures at the nanometric scale, with intentionally introduced tunable “decorative impurities” or well-defined interfaces forming bilayer structures. These groups of modified or functionalized structures, in which metal oxides are still the main protagonists either as host or guest elements, have proved improvements in VOCs sensing. The work also identifies the need to explore new hybrid material combinations, as well as the convenience of incorporating other transducing principles further than resistive that allow the exploitation of mixed output concepts (e.g., electric, optic, mechanic).

## 1. Introduction

Nanoscaled materials, with sizes within the Debye length of the surface (typically on the order of 2–100 nm), are used as highly sensitive (receptor) elements in gas/vapor sensors due to their large surface-to-volume-ratio and their proven better chemical, optical, and electrical properties as compared to non-nanoscaled materials. These gas/vapor sensitive materials include usually unmodified or modified metal oxides (MOXs), polymers (POMs), and/or carbon-based materials (CbMs) with varied morphological shapes, integrated into conventional transducers (e.g., resistive, capacitive, gravimetric, or optical). The sensing properties of these materials generally depend on the chemisorption of negatively charged oxygen adsorbates (O^−2^, O^−^, and O^2−^) in air, which, due to charge transfer between the material and the analytes, change the electron density on the material surface. They may also depend on the chemical reaction between the sensitive material and the analyte, or the diffusion of species into the bulk of the material [1,2,3,4,5,6,7,8,9,10].

Most studies of gas/vapor sensitive materials are focused on the detection of low concentrations of CO, NO_2_, O_3_, H_2_, NH_3_, or H_2_S due to their toxicity, their relation with atmospheric composition, or the fact that these gases can be found at high levels in certain environments [11,12,13,14,15,16,17,18]. However, in recent years, the application of gas/vapor sensitive materials has been extended to the detection of volatile organic compounds (VOCs), not only because their presence is significant in the industry and domestic sector, but also because of their relevance as markers for (indoor/outdoor) air [19] and food quality [20], and early diagnosis of several diseases [21].

VOCs are organic chemicals that possess a high vapor pressure at room temperature [22]. These compounds are numerous and varied, commonly classified according to their functional group into aliphatic hydrocarbons, simple oxygenated hydrocarbons, halogenated hydrocarbons, carbonyl compounds, aromatic hydrocarbons, etc. [23]. Most applications, in which one or more of these compounds need to be monitored, typically involve a broad background of other vapors and/or gases. For instance, thousands of VOCs have been found in the environment, food products, and/or exhaled breath. Therefore, the selectivity of sensitive materials, or its approximation by means of reducing the cross-sensitivity, is imperative. Table 1 exemplifies the VOCs profile in the field of air quality and breath analysis (note that these profiles contain only the most representative VOCs usually reported in the literature). 

**Figure 1:**
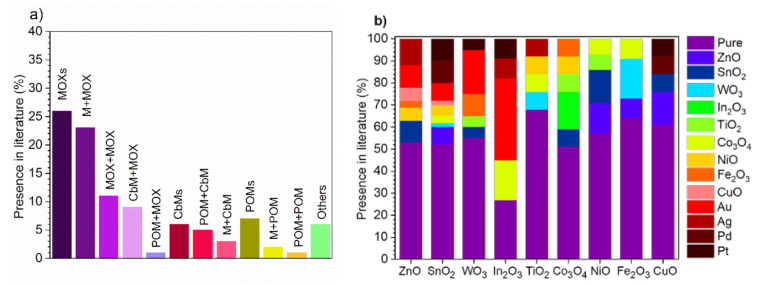
Materials for VOCs sensing summed up from the Web of Science (WOS) database. The data were collected using “gas sensors + VOCs” as keywords and only journal articles between 2015 and 2020 were taken into account. The materials were generalized as modified metal oxides (MOXs) (e.g., ZnO, SnO_2_, WO_3_), carbon-based materials (CbMs) (e.g., graphene, graphene oxide, carbon nanotubes), and polymers (POMs) (e.g., conductive polymers and others such as Poly(methyl methacrylate)—PMMA). (**a**) Summary of MOXs, POMs, and CbMs, plus their mutual modifications (MOX + MOX, CbM + MOX, POM + MOX, POM + CbM, POM + POM) or metal modifications (M + MOX, M + POM, M + CbM), and other minority materials (e.g., zeolitic imidazolate framework (ZIF) materials, metal dichalcogenides, and perovskites) used for VOCs sensing. (**b**) List and presence in the literature of the most common VOC sensitive MOXs (pure) and their modifications with metals or another MOX. Notice that the literature provides further examples of single and complex MOXs (based on elements from the transition, post-transition, lanthanide, and alkaline earth groups from the periodic table) than those presented here, but these examples are comparatively less recurrent in VOC sensing.

**Figure 2:**
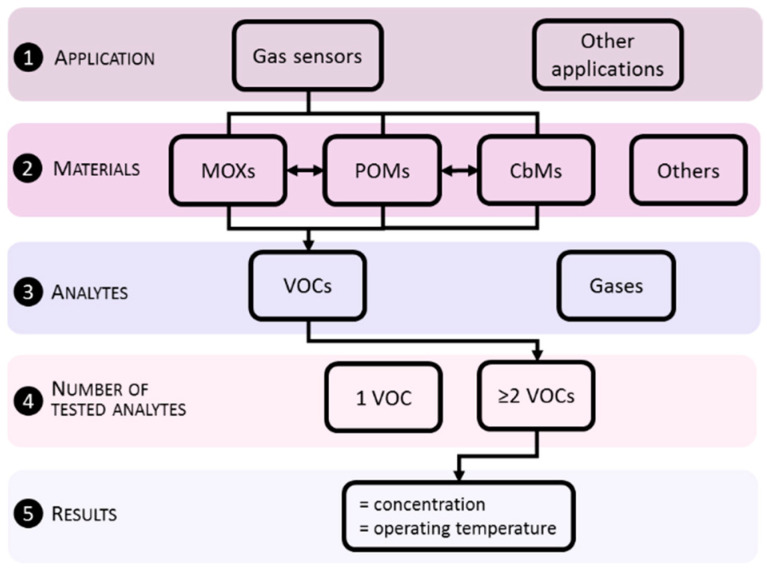
Schematic representation of the filtering stages used as criteria to search the literature reports for this review, in particular those displayed in the tables (4 to 9). The stages include a selection by application, materials, analytes, and number of analytes tested, with the results limiting the data to those reports in which two or more VOCs are evaluated using the same operating temperature and concentration for each analyte.

The present review focuses on the materials utilized to improve VOCs sensing, paying special attention to properties such as the sensitivity, the response time, and particularly selectivity from a cross-sensitivity point of view. The key materials taken into account in this review are MOXs, POMs, and CbMs and their modifications, with a special effort dedicated to MOXs due to their large presence in the literature, even after several years of research in this field. This can be noticed in Figure 1a, which displays an example of the weight of reports for these materials in the recent five years, as well as the most common unmodified and modified MOXs (Figure 1b). In considering the literature, we have compiled extensive tables with selected sensing data. A schematic view of the criteria used for such selection is depicted in Figure 2. The filtering stages have focused on finding reports in which the targeted materials were tested to various VOCs at the same concentrations and operating temperatures to compare their crossed response. This search sorting has significantly reduced the number of reports as compared to the comprehensive list of available literature, which certainly provides extraordinary examples of materials and their analysis, but often these publications do not cover in depth the test of analytes or in many cases, they present responses to incomparable analytes concentrations or operating temperatures.

In summary, the tables provide to the reader a quick visualization of the best responses for a given material and the degree of cross-response recorded at the same operating temperature for similar concentrations of various VOCs. The tables showing functional parameters, although providing quantitative data, are recommended to be used only for qualitative comparison, as the reports do not share generalized protocols for the tests. Thus, the functional outputs are prone to not depend only on the material properties, but also on the conditions used to test the analytes. The review, in addition, provides the most common technological solutions reported in the literature, including material features, synthetic methods, sensing principles, and the key enabling systems for their practical use. We believe this work will allow for identifying at one glance the attributes of most common systems for VOCs sensing, among MOXs, POMs, and CbMs in terms of cross-response amongst selected VOCs; information generally not found in other reviews. Hence, here we present the recent research efforts and developments in materials for VOCs sensing.

## 2. Gas/Vapor Sensitive Materials

The gas/vapor sensitive receptors usually include materials with semiconducting properties such as MOXs, POMs, and CbMs, although, amongst them, MOXs are the primary materials used for gas/vapor sensing. MOXs work as sensitive materials due to their properties to adsorb gas/vapor chemicals and transduce them into conductivity changes. These properties were exploited for the first time in the 60s by Seiyama et al. to fabricate ZnO based chemoresistive gas sensors [31]. Since then, a vast number of MOXs have been studied and addressed to enhance their sensing features, taking into account their electro-physical (band gap, electroconductivity, type of conductivity, oxygen diffusion), thermodynamic, surface, electronic, structural, and catalytic properties [3,32]. Thus, the literature [33] demonstrated that MOXs with large band gaps (e.g., between 2 and 4 eV) and low activation energy of the centers are generally an optimal combination for chemoresistive sensors. In addition, the studies on various n- and p-type MOXs pointed out that the most effective and widely used MOXs are those with n-type conductivity, such as ZnO, SnO_2_, and WO_3_ [33,34]. Certainly, this is consistent with Figure 1b, which suggests that these three materials are amongst the dominant gas/vapor receptors in the literature. ZnO, for instance, is an n-type semiconductor with a direct band gap of 3.37 eV [35] which has shown large surface-to-volume ratio morphologies that improved sensitivity and response speed with respect to other morphologies with bulky characteristics. This material showed especially good sensitivity to methanol [36], ethanol [37,38], chlorophenol [36], formaldehyde [39], and acetaldehyde [39,40]. The wide use of ZnO in the literature is probably related to its relatively facile synthesis processes and high chemical stability [41]. SnO_2_ is another n-type gas/vapor sensitive MOX with wide band gap of 3.6 eV [42]. This material has been used intensively for studying the influence of grain size on sensor response [2,43], the conductivity of MOX gas sensors [1], or the key surface properties for gas sensing [3], amongst others. SnO_2_ has shown good mechanical stability, high conductivity, wide operating temperature range (200–600 °C) [44] and excellent performance in the detection of ethanol [45], butanol [46], acetone [45], and toluene [47]. Other important gas/vapor sensitive MOXs include WO_3_, In_2_O_3_, TiO_2_, and Fe_2_O_3_. Specifically, WO_3_ showed high sensitivity to VOCs such as acetone [48], methanol [48], and toluene [49]. Moreover, In_2_O_3_ exhibited very high responses to acetone and toluene with a detection limit of 500 ppb [50] and TiO_2_ demonstrated very promising results in acetone [51] and hydrogen [52] detection, while Fe_2_O_3_ nanoparticles were used for the decoration of common MOXs (e.g., WO_3_ or SiO_2_) showing high sensitivity to toluene [49] and ethylacetate [53].

POMs are organic materials usually belonging to the category of insulators, as they possess covalent bonds without free movable charge carriers (electrons or ions), although within POMs there is a group of conductive polymers, which behave similarly to semiconductor MOXs. Their conductivity originates from highly delocalized electrons between the conjugated polymer backbone (alternating single bonds (σ) and double bonds (π)) and the dopant (negatively charged species). When conductive polymers are exposed to the gas/vapor, these materials undergo oxidation and reduction processes as a function of the dopants (p- or n-type) [4]. During the oxidation, electrons are extracted from the highest occupied molecular orbital (HOMO) of the valence band, whereas during the reduction, electrons are transferred to the lowest unoccupied molecular orbital (LUMO) of the conduction band. The charge carriers in the form of polarons, bipolarons, or an equilibrium between both are created during the redox processes [5,6]. This charge transfer is generally transduced in conductivity changes that enable the gas/vapor detection even at room temperature, circumventing the need for thermal activation as in MOXs. Conductive polymers such as polypyrrole (PPy), polyaniline (PANI), polythiophene (PTh), and poly(3,4-ethylenedioxythiophene) (PEDOT), and their modifications with metals have been the most studied candidates for sensing VOCs [4,7] (Figure 1a). These materials demonstrated mostly resistive responses to vapors such as ethanol, methanol, and acetone, and gases such as NH_3_, NO_2_, and CO [5]. Macrocyclic compounds such as cyclodextrins (CDs), calixarenes, and cavitands have also been investigated due to the presence of cavities with molecular dimensions, which can act as molecular receptors [8].

CbMs include mainly carbon nanotubes (CNTs), graphene oxide (GO), or reduced graphene oxide (rGO). Like other gas-sensitive materials, it is widely accepted that the charge transfer between gas/vapor species and CbMs is responsible for the conductivity changes of these materials and thus gas/vapor detection. Due to the typical two-dimensional (2D) single-atom-thick structure of graphene, GO and rGO, these materials are prone to change the local carrier concentration electron by electron providing these structures advantages over CNTs and other MOXs and POMs [9,10]. Overall, the gas sensing properties of CbMs are strongly connected with the presence of defects and residual contaminants at the surface. Hence, for practical applications, the sensitivity of CbMs is usually enhanced by modifying or functionalizing them with metals [54,55], MOXs [56], or POMs [57,58], as can be seen in Figure 1a.

Overall, the key sensing features pursued in gas/vapor sensitive materials include sensitivity, selectivity, stability, and speed of response; features known as the four “S”s of gas sensing. Moreover, these materials are expected to interact reversibly and reproducibly with the specific analyte of interest showing strong stability in harsh conditions such as high temperature and/or high relative ambient humidity. From the economical point of view, the sensitive material should be widely available, simply and inexpensively synthesized, compatible, and easily implemented with specific transducing platforms. Table 2 presents a summary of the possible advantages and limits of these materials in their unmodified form. This table is intended to provide only a qualitative view, the quantitative evaluation for these materials is provided in the next sections.

Despite the great efforts invested in solving the mentioned limitations for each material type, and the large amount of literature showing important improvements, there is still a continuous demand in the gas sensor field for enhanced materials properties. The pursued improvements are especially focused on finding materials with more sensitivity and selectivity to VOCs from the aliphatic and aromatic hydrocarbons, and carbonyl groups, rather than just those from the simple oxygenated hydrocarbons group, as well as lower detection limits that reach ppb-ppt levels. The demands also emphasize the search for scalable synthetic methods for on-chip integration and low operational power consumption. Currently, the most common strategies to pursue these improvements at the material level generally involve tailoring of the material by:

- Tuning the surface morphology: shape, size, and dimensional control to obtain 0D (e.g., nanoparticles and quantum dots), 1D (e.g., nanofibers, nanotubes, and nanowires), 2D (e.g., thin films, nanosheets, and nanoplates), and 3D (e.g., porous films and nanoflowers, which consist of 2D nanosheets) materials.
- Modifying or functionalizing the material: control of type and level of intentional impurities (doping, formation of Schottky barriers, heterojunctions, and/or inorganic/organic hybrid structures).

## 3. Tailoring Materials for Enhanced Sensing Properties to VOCs

### 3.1. Surface Area—The Larger, the Better

Several works in the literature have stated the correlation between the sensing performances, morphology, microstructure, and size of gas/vapor sensitive materials [59,60,61,62,63,64]. In this context, various materials with morphological shapes in the nanometric scale with increased surface-to-volume ratio have proven to be more effective for gas detection [46,47]. Generally, the surface properties of these peculiar morphologies, including crystalline and structural properties, have been shown to determine the sensing activity of these materials. More precisely, the density of states at the surface has been revealed to play an important role in the sensing mechanism of gases and vapors dominated by the space-charge-controlled grain-boundary conduction model, in which the grain sizes, surface chemical states, and gas/vapor adsorption amount determine the overall response of the sensors [1].

**Figure 3:**
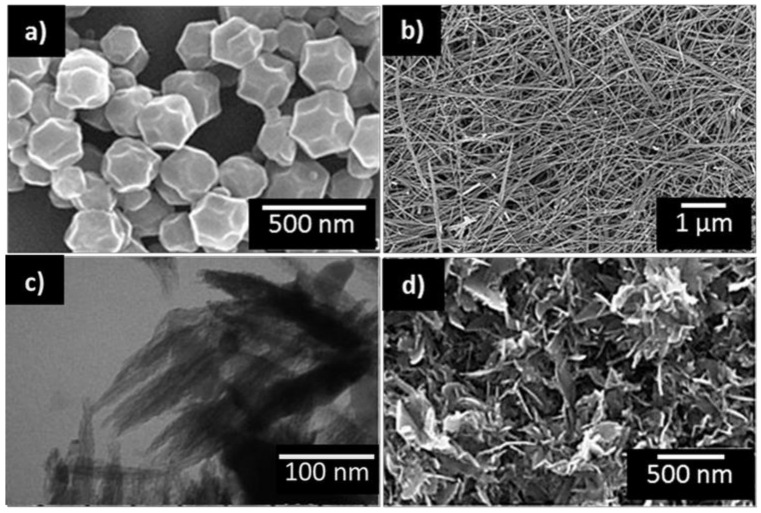
SEM images of MOX nanostructures with different morphologies and dimensionalities (**a**) 0D—cubes, (**b**) 1D—needles, (**c**) 2D—sheets, and (**d**) 3D—flowers. Adapted from references [39,69,75], with permission from the American Chemical Society, 2014, and Elsevier B.V., 2013 and 2016, respectively.

Table 3 displays a collection of low dimensional and 3D nanomaterials that have proved greatly improved functionality compared to bulk materials for VOCs sensing. Examples of the appearance of selected morphologies are also displayed in Figure 3. Certainly, MOXs, and in particular SnO_2_, have been the primary reference materials for determining the influence of surface area (particle size) on gas sensing properties in the past [2,43]. The literature related to VOCs, for instance, shows that SnO_2_ nanoparticles with reduced crystalline size (from 9.1 to 6.1 nm) show higher and faster response to butanol [46] and that non-commercial nanoparticles can be improved for obtaining better responses to toluene [47] (Table 4). Similarly, other comparative studies of sensors based on complex structures with flower-like hierarchical porous single-crystalline ZnO nanosheets and commercial ZnO powder demonstrated for the former three-times better responses to ethanol with nearly 40% faster response [37]. This fact brought attention to further studies, which focused on controlling the features of various ZnO nanostructures [40]. The results demonstrated enhanced sensitivity to acetaldehyde in the sub-ppm range (50 ppb) when tuning the morphology into nanorings shape instead of nanoparticles, nanorods, or nanotubes.

Other studies discussed the impact of the dimensionality of TiO_2_ nanostructures on the gas sensing performance. For instance, hierarchical 3D TiO_2_ nanoflowers [51] showed enhanced properties towards acetone at room temperature with respect to TiO_2_ nanoparticles [77]. The use of hierarchical flower-like CuO nanostructures is another example of complex 3D-shaped MOX [74], which has reported excellent results towards ethanol, formaldehyde, acetone, and xylene for concentrations as low as 20 ppb and yet with fast response (11.2 s) and short recovery time (8.4 s). Novel MOXs based on highly porous Co_3_O_4_ concave nanocubes with high surface area (120.9 m^2^g^−1^) have also proven fast response and recovery time below 10 s to 10 ppm of ethanol [69]. Similarly, Co_3_O_4_ in the form of monolayer has shown high responses to ethanol and xylene [72]. Other complex structures, for instance, nanosheet-based 3D hierarchical rosette-like In_2_O_3_ microspheres in their hollow and full form, also showed promising properties to low (500 ppb) acetone and toluene concentrations [50]. Recently, a new study [76] reported the use of ZnO nanotetrapods, consisting of four cylindrical nanorod legs, which showed good results in the detection of VOCs, especially ethanol. Although there is no general rule to state a better sensing functionality for one or another morphological dimension [78], the literature compiled for this review suggests the 3D morphology to be superior in gas/vapor sensing compared to 0D, 1D, and 2D. It is worth bearing in mind that 3D structures are assembled from low dimensional (0D, 1D, or 2D) nano-building blocks and therefore further improvements might be related to synergistic effects [79]. The apparent improved behavior in 3D structures could also relate to their particular surface defects due to their porous and irregular arrangement. In addition, the interconnected structure, usually present in 3D-based films, seems to provide further advantages, especially in resistive-based sensors (the most representative in the literature of nanomaterial-based sensors) as it favors the electrical current percolation [80,81]. This is the opposite for other types of structures, for instance, those based on vertically aligned 1D structures. Thus, the selection of the materials by their dimension should not be associated exclusively with the surface-to-volume-ratio feature, but rather with the employed working principle and the targeted transducing platform.

The reports in the literature are not limited only to single oxides; the morphology control of multicomponent oxides as well as the chemical composition for sensing VOCs have also shown to improve the sensing performance. For example, large surface nonstoichiometric Co-rich ZnCo_2_O_4_ in the form of hollow nanospheres reported high sensitivity to formaldehyde with superior experimental and estimated detection limits of 13 ppb and 2 ppb, respectively, as compared to other reports in the literature [70].

POMs and CbMs with large surface areas are also pursued to enhance VOCs detection. However, in the gas/vapor sensing literature, there is a lack of information about the size influence on POMS and CbMs as frequently the reports regard the functionalization effect on these materials, which indeed have proven essential to promoting VOCs sensitivity; this is further discussed in the next section. Most of the reports on pristine POMs and CbMs state high sensitivity to gases such as NO_x_ and NH_3_, and nearly insensitivity to VOCs especially from the aromatic family [4,57,82,83,84]. Among the few examples of VOCs sensing with pristine CbMs materials (Table 4), we found that using pristine single-walled carbon nanotubes (SWCNTs) instead of multi-walled carbon nanotubes (MWCNTs) might be favorable to detect toluene at room temperature [85].

Besides MOX- and GO-based 2D materials, it is important to mention other emerging materials with the same dimensionality that have a significant role in VOCs detection, although not the scope of this review. Among them are 2D transition metal dichalcogenides (TMDs) with their unique physical and chemical properties, such as a high surface-to-volume ratio, large number of active sites for redox reactions, high surface reactivity, and high absorption coefficient [86]. These materials have the formula MX_2_, where M stands for transition metal element (e.g., Ti, Zr, Hf, V, Nb, Ta, Mo, W, Re, etc.) and X represents a chalcogen (Se, S, or Te) [87]. Some of the most common TMDs for VOCs detection are MoS_2_, WS_2_, MoSe_2_, WSe_2_, and SnS_2_ [86,88,89,90,91].

Within the novel 2D materials, transition metal carbides/nitrides (MXenes) represent another important group. These materials have stable and easily tunable microstructure, high electrical conductivity, large chemically active surface, and adjustable hydrophilicity, which makes them good candidates for VOCs monitoring [86]. The most studied MXenes for VOCs detection are Ti_3_C_2_Tx, V_2_CT_x_, Mo_2_C, and Ti_2_CO_2_ [92,93]. Another noteworthy 2D material is boron nitride (BN). The high sensitivity reported for BN toward VOCs is related to localized charges induced by the presence of B and N atoms [94]. Amongst 2D materials, there are also those that are constituted of just one atomic species such as one layer thick black phosphorus (phosphorene), silicon (silicene), or germanium (germanene) [95]. Black phosphorus (BP) is considered to be a promising alternative [96,97], since compared to graphene and TMDs, BP possesses a higher surface-to-volume ratio due to its puckered honeycomb lattice structure [98,99].

### 3.2. Modification or Functionalization—Pushing the Performance Further

**Figure 4:**
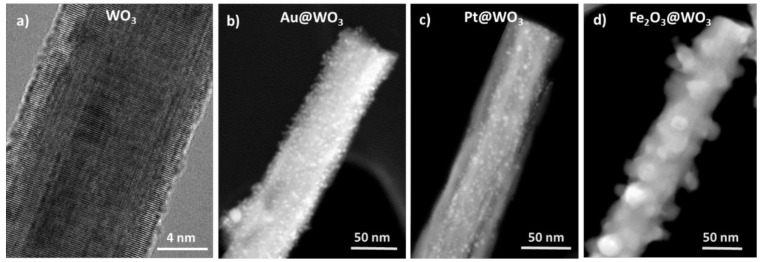
TEM images of (**a**) unmodified (high-resolution TEM) WO_3_ nanowire and modified (scanning TEM) with (**b**) Au, (**c**) Pt, and (**d**) Fe_2_O_3_ nanoparticles.

Another strategy commonly used to improve further the sensing properties of materials towards VOCs consists in the incorporation of intentional “impurities” at/in the material surface/structure. This modification or functionalization process includes the so-called:

- Decorated materials, when incorporating low amounts of noble metals or secondary materials (e.g., MOXs, POMs, CbMs) at the surface. These are represented by an “@” sign in between the guest and host material, e.g., Au@WO_3_. Figure 4 displays examples of the decoration of WO_3_ nanowires with gold, platinum, or iron oxide.
- Simple mixtures, when mixing two or more gas sensitive materials randomly. These are represented by a hyphen “-“ sign in between the constituents, e.g., ZnO-CuO.
- Bilayers and trilayers, when there is a well-defined partition or interface between the two or three gas sensitive materials. These are represented by a slash “/” sign in between the constituents, e.g., CuO/SnO_2_ and GO/ZnO/GO.
- Doped materials, when incorporating “guest” atoms into the material structure, e.g., Ce-doped SnO_2_.

Hereafter, the review differentiates the functionalized materials by the nomenclatures exposed above. The enhanced sensing performance of these functionalized materials has been attributed to electronic effects (band bending due to Fermi level equilibration, charge carrier separation, tuning of the depletion layer, and increased interfacial potential barrier energy) and/or chemical effects (decrease in activation energy, targeted catalytic activity, and synergistic surface reactions). The functionalization in terms of doping and decoration by forming nano-Schottky barriers or heterojunctions has been discussed broadly in previous studies [32,100]. Results in the literature [60,101] show that functionalized materials improve further the sensitivity, response time, working temperature, and/or selectivity to some extent by reducing the cross-responses to different gas or vapor molecules. In this work, we bring attention to the “decorative” and doping materials that have improved MOXs, POMs, and CbMs based sensors towards VOCs detection (Table 5).

The number of entries in Table 5 demonstrates that the decoration of popular gas-sensitive MOXs (SnO_2_, ZnO, WO_3_) with noble metals (e.g., Au, Pt, Pd) or secondary oxides (ZnO, TiO_2_, CuO, Fe_2_O_3_) is a generalized method for improving the sensing performance of MOXs towards VOCs. For instance, research on the load variations of Au at ZnO revealed the dependence of the response on Au concentration. Results showed that Au-loaded ZnO has better responses to various VOCs, including diethylether, acetone, chlorobenzene, trichloroethylene, isoprene, ethylacetate, butylacetate, formaldehyde, and hexaldehyde. The 3 at.% Au@ZnO systems improved the response to isoprene, while the 5 at.% Au@ZnO to methanol [65]. Similarly, investigations on Ce- and Mg-doped SnO_2_ found better sensing performance to formaldehyde, methanol, ethanol, and acetone for 2% Ce-doped SnO_2_ and ethanol, toluene, and acetone for 1 at.% Mg-doped SnO_2_ as compared to unmodified SnO_2_ [73]. Further, the decoration of TiO_2_ nanorods with 12 at.% of Pd showed enhanced responses to isopropanol as well as a clear improvement to hydrogen, which registers 35 times better response for the Pd@TiO_2_ systems than for the unmodified TiO_2_ nanorods [52].

The functionalization of MOXs with secondary MOXs, instead of noble metals, also revealed further advantages. For instance, the sensing properties of WO_3_ nanowires decorated with Fe_2_O_3_ nanoparticles to toluene proved to be comparable to Pt@WO_3_ nanowires, indicating that the use of expensive precious metals for MOXs functionalization could be circumvented [48,49]. Other examples of secondary MOXs include CuO, which was employed to improve the sensing properties of ZnO to acetone [75] and SnO_2_ to xylene [45]. This type of modified system (i.e., MOX/MOX) also showed advantages in cataluminescence (CTL) gas/vapor sensors. Thus, TiO_2_/SnO_2_ exhibited better CTL properties including stable intensity, high signal/noise values, short response, and better sensitivity to benzene and toluene compared to intrinsic SnO_2_ [102].

The modification of CbMs and POMs with inorganic materials, mainly MOXs and noble metals for improving their VOCs sensing properties is also frequent in the literature (Table 5). In this context, results on the use of Pt and Pd to decorate MWCNTs are of great interest since these modified materials enabled response to non-aromatic VOCs, in particular methanol [54]. Further studies in the same context showed that the array of oxygen treated MWCNTs and FeO decorated MWCNTs allows for distinguishing the presence of aromatic VOCs such as toluene and benzene in a background of non-aromatic VOCs [104]. Another example of CbMs modification involves NiO/MWCNTs [55]. This composite material exhibited higher response than unmodified NiO nanoparticles to VOCs, especially ethanol. Authors attributed these improvements to the increased surface area and extra interfaces in NiO/MWCNTs, which offer more active sites for the adsorption of VOC molecules and facilitate to get over the energy barriers as well as reducing the operating temperature. CbM_S_ have also been used as modifiers, for instance, low amounts (0.1 wt.%) of MWCNTs on ZnO hollow spheres matrix enhanced dramatically the response of ZnO to VOCs, mainly acetone [106]. These experiments also determine that the content of MWCNTs defines the optimum operating temperature and degree of response enhancement to VOCs, allowing for a modulation of the cross-response among the targeted VOCs.

The reports related to POMs modification also point out improved functionality by their combination with other materials. In this line, a study of nanocomposites in which small amounts of Au [113] or Ag [107] were embedded into PPy showed improvement in the sensing properties of PPy to low ethylene and acetone concentrations, respectively. A similar approach also indicated potential advantages on acetone detection for PPy modified with CdTe quantum dots [108] and polytetrafluoroethylene (PTFE) modified with Ti [109]. The modification of PEDOT with a low amount of GO also proved a higher response for PEDOT to methanol at room temperature. Similarly, CD incorporation in PANI enhances the sensor efficiency of pristine PANI to benzene and toluene detection [68].

Further modifications on gas/vapor sensitive materials include the use of multicomponent such as Ag/SnO_2_/GO [110] or multilayers of GO/ZnO/GO [111], although their performances were not proven to represent additional advantages with respect to traditional modified materials with two components.

## 4. Selectivity—In Search of Specificity

Another important consideration in gas/vapor sensitive materials relates to their selectivity. In gas/vapor sensors, the selectivity refers to the ability of the system to identify (discriminate) a particular gas/vapor analyte in a complex mixture, avoiding interfering responses from other analytes, with the ultimate achievement of specificity [114]. However, in practice, a specific response is unrealistic, and thus most of the analysis of gas/vapor sensitive materials deal with selectivity, and usually, straightforward comparative studies of responses to various analytes. Table 6 and Table 7 summarize these findings so that the reader can distinguish the degree of cross-response recorded for a given material and group of VOCs in selected reports.

VOCs are numerous, varied, and ubiquitous. The list of these compounds amounts to more than 1000 species [115]. However, only a few of these are recurrent in gas sensing studies (e.g., ethanol, methanol, acetone, benzene, toluene, formaldehyde, or acetaldehyde) probably because of their abundance in the industry and the indoor/outdoor environment, and their significance as biomarkers in disease diagnosis, as summarized in Table 1. Therefore, Table 6 and Table 7 display a list of over 30 VOCs, which correspond to the usual compounds analyzed by the gas sensors community. The higher data density for ethanol, methanol, and acetone compared to the other compounds evidences the special attention on such vapors. The tables also include other gases of interest (e.g., ammonia, hydrogen, nitrogen dioxide, carbon monoxide, and hydrogen sulfide) commonly used in the cross-response tests performed in the literature. The bottom and top of the table display the gas-sensitive materials and the “optimum” operating temperatures for the concentration annotated in each referenced material, respectively. The color codes from blue to red and numbers from 1 to 10 along each column show the tested VOCs and their increasing load of response with an increasing number for each referenced material; note the responses are compared for the same VOCs concentration in each case. The numbers from 1 to 10 are calculated so that the highest gas response (for one specific material) is marked with the number 10, while the responses from other gases are compared to that one. This calculation was repeated for each reference (each column) separately. Thus, for instance, in the first column of Table 6, one can notice that porous ZnO film synthesized by hydrothermal reactions showed the highest responses to methanol (red 10) with a high probability of interference from other VOCs such as 2-chlorophenol (brown 9), acetone (orange 8), and 2-chloroethanol (green 6) and moderate interference from VOCs such as formaldehyde (turquoise 3), and chlorobenzene (blue 1) [36].

It is worth noting that most of the gas-sensitive materials in Table 6 and Table 7 rely on the resistive transducing principle (Table 8 and Table 9 highlight this in more detail). For those reports using other approaches (e.g., cataluminescence or surface acoustic waves), the discussion includes a specific remark on this aspect. A couple of examples on the cross-response of Au and Ag nanoparticles using localized surface plasmon resonance is also included, due to the potential use of this transducing principle on MOXs, POMs, and CbMs modified with plasmonic nanoparticles. Hereafter, we sum up the VOCs interfering patterns by their functional group classification.

- Simple oxygenated hydrocarbons

Simple oxygenated hydrocarbons are hydrocarbons that contain oxygen as a part of their chemical structure. These compounds involve species such as alcohols (e.g., ethanol, methanol, butanol, to cite a few), and ethers (e.g., diethylether and tetrahydrofuran).

The selectivity to **methanol** is apparently favored by using Pt-decorated WO_3_ nanoneedles [48] and localized surface plasmon resonance (LSPR) Au nanoparticles-based sensors [66], which have shown moderate interference to ethanol with promising low cross-responses to acetone, toluene, and isopropanol. Another possible option for high sensitive methanol materials includes ZnO porous films [36], although acetone and 2-chlorophenol may interfere with methanol response. Among organic materials, poly(3,4-ethylenedioxythiophene)-poly(styrenesulfonate) modified with ultralarge graphene oxide (PEDOT-PSS/UL-GO) showed excellent selectivity to methanol in front of vapors such as ethanol, acetone, propanol, toluene, formaldehyde, and water [57]. MWCNTs decorated with Pt or Pd worked successfully for methanol detection too proving low cross-sensitivity to acetone, although with high interference to ethanol [54]. The combination of ZnO hollow spheres with 0.05 wt.% MWCNTs [106] also showed improved selectivity to methanol with moderate interference from ethanol and much lower interference from acetone and ether. Additionally, core-shell hybridized Fe_3_O_4_ magnetic nanoparticles synthesized in the presence of polymerized ionic liquids and modified with poly(3,4-ethylenedioxythiophene) derived from poly(ionic liquid) (PEDOT-PIL@Fe_3_O_4_) [116] suggested advantages to discriminate methanol from benzene and toluene, but with high interference from ethanol and acetone.

Generally, the interference patterns in Table 6 and Table 7 for oxygenated hydrocarbons such as **ethanol** indicate the best selectivity to this compound with only minor interferences from acetone and toluene by using flower-like hierarchical structures consisting of porous single-crystalline ZnO nanosheets [37], porous Co_3_O_4_ concave nanocubes [69], or ZnO nanotetrapods [76]. Another alternative for ethanol detection, although with moderate interferences from acetone and low interference from isopropanol and toluene, was achieved by electrochemically synthesized ZnO porous films [38]. SnO_2_ obtained by microwave-assisted approach [45] has shown to be a less discriminating material for ethanol in the presence of acetone. However, this material shows the potential to discriminate either ethanol or acetone in the presence of various aromatic compounds (e.g., benzene, toluene, and xylene) and formaldehyde. In contrast, hierarchical flower-like CuO nanostructures [74] have shown high ethanol responses, though with high interferences to acetone, xylene, and formaldehyde. CbMs such as MWCNTs decorated with Au also showed a good response to ethanol with possible interference from benzene [55]. The addition of MHDA to this composite (i.e., MWCNTs/Au) improved further the selectivity to ethanol, reducing the interference to benzene. This implies that the functionalization with MHDA improves discrimination by boosting sensitivity to non-aromatic VOCs and suppressing the response to aromatic VOCs [55]. The use of composites formed by rGO and SnO_2_ also proved to be appropriate to distinguish ethanol among aromatic VOCs, such as benzene, toluene, and xylene with moderate interferences from methanol [112].

The reports in Table 6 and Table 7 also show that the detection of **propanol** has been favored by the use of modified materials such as Ag-BC-bsh [117] and Ni/MWCNTs [105]. However, these materials present high cross-responses with other oxygenated hydrocarbons and eventually with ammonia. Further studies suggest the selectivity to **butanol** among ethanol, methanol, acetone, benzene, toluene, and formaldehyde by using mesoporous worm-like SnO_2_ films [46]. Similarly, an improved selectivity to **pentanol** with respect to octane and toluene could be achieved by Ag nanoparticles with LSPR, bearing in mind possible high cross-sensitivity to xylene [67].

Improved properties to **ether** were achieved using La_2_O_3_ and SiO_2_/Fe_3_O_4_ nanoparticles, both indicating very low interferences with tetrahydrofuran, alcohols, acetone, benzene, aldehydes, and halogenated hydrocarbons [53]. Results for **diethylether** also suggested good selective detection of this VOC among acetone, heptane, decane, benzene, toluene, and xylene, with partial selectivity to methanol, hexaldehyde, chlorobenzene, and high interferences to isoprene and ethylacetate.

- Aliphatic hydrocarbons

Aliphatic hydrocarbons are present in several environmental and industrial sectors, with methane as the most representative compound among them. The track of results on aliphatic hydrocarbons using MOXs, POMs, or CbMs is not extensive, most likely influenced by the low sensitivity often reported for semiconducting materials to these compounds. This fact is visible in Table 6 and Table 7 that display a low amount of reports, in which aliphatic compounds registered higher responses in respect to other VOCs or toxic gases. Among the few reports, one can see a relatively good selectivity to **isoprene** by using porous single-crystalline ZnO nanoplates decorated with Au NPs [65]. These records suggest low interferences of isoprene with heptane, decane, acetone, formaldehyde, trichloroethylene, and aromatic compounds including benzene, toluene, and xylene, as well as high interferences, particularly to ethylacetate and diethylether. Recently, hierarchical hollow SnO_2_ spheres modified with Pt have also shown potential to detect **methane**, although their performances in terms of selectivity to compounds such as ethanol, benzene, formaldehyde, and ammonia appear to be unviable as their cross-responses are reduced only when the methane concentrations are hundreds of ppm (>250) higher than the concentration of the other compounds [118].

- Aromatic hydrocarbons

Aromatic hydrocarbons are generally associated with compounds containing the benzene ring. These compounds exhibit aromaticity, most of them characterized by sweet or pleasant odor. The reports on sensitive materials for aromatic hydrocarbons are not as extensive as those for oxygenated hydrocarbons. In fact, a search of the literature in terms of their selectivity indicates frequently poor load of response for these compounds among oxygenated hydrocarbons, as well as esters, ethers, and aldehydes.

According to Table 6 and Table 7, the strongest selectivity to **benzene,** in front of various alcohols, and other aromatic compounds such as toluene and xylene, were recorded for modified La_2_O_3_ nanorods with Au nanoparticles [119] and coral-like TiO_2_/SnO_2_ porous film [102] implemented using CTL concept. Partial selectivity to benzene was also noticed for MWCNTs decorated with Au nanoparticles [55], especially in comparison to acetone. Materials based on modified Fe_2_O_3_/WO_3-x_ nanoneedles [49] and porous flower-like SnO_2_ nanosheets [47] also appear to be a good option to attain partial selectivity to **toluene** among ethanol, methanol, and acetone. However, the cross-response to benzene and formaldehyde could still be an issue. As for **xylene**, the selectivity to this compound among acetone, methanol, isopropanol, and chloroform seems to be greatly improved for Pd-functionalized ZnO nanorods [71] integrated into quartz crystal microbalance (QCM). Results for CuO/SnO_2_, in contrast, show higher interferences for xylene in front of alcohols and other aromatic compounds, though the interferences could be reduced towards formaldehyde and ammonia. In the same gas family, a strong selectivity to xylene among ethanol, benzene, formaldehyde, and carbon monoxide has been reported using monolayers of periodic porous Co_3_O_4_ inverse opal thin films [72]. Although calixarene functionalized SWCNTs has shown good potential for xylene detection, high interferences from toluene and ethylbenzene are noticed [120].

- Carbonyl compounds

Carbonyl compounds can be also classified as oxygenated hydrocarbons, since they contain oxygen atoms in their structure, but unlike simple oxygenated hydrocarbons, carbonyl compounds come from carboxylic acids. This group includes ketones, aldehydes, carboxylic acids, acid anhydrides, esters, amides, and acid halides.

As far as **acetone** detection is concerned, the reports indicate a strong selectivity to this compound among methanol, butanone, isopropanol, and toluene for hierarchical 3D TiO_2_ nanoflowers operating at low temperature (60 °C) [51]. Other materials, based on unmodified or Au-modified WO_3_ nanoneedles [48], also point toward good selectivity to acetone among ethanol, methanol, and toluene, contrary to Ce-doped SnO_2_, which revealed higher interferences for acetone among methanol, ethanol, and formaldehyde [73]. The use of a mixture based on ZnO-CuO has also shown the potential to discriminate acetone among ethanol and typical toxic gases including ammonia, nitrogen dioxide, and hydrogen sulfide [75]. In_2_O_3_ [50] and Y_2_O_3_ (with CTL-based signal) [53] present themselves as other unmodified MOXs with prospective partial selectivity to acetone, the first suggesting good or moderate discrimination from methane, formaldehyde, and chloroform, whereas the second showing good discrimination from various alcohols, aldehydes, and aromatic compounds. ZnO/MWCNTs hybrid material with MWCNTs content of 0.1 wt.% also showed to be selective to acetone among ethanol, methanol, and ether [106]. Other materials such as metal-organic frameworks derived hierarchical hollow ZnO nanocages (MOF-ZnO) displayed good acetone discrimination among ethanol, benzene, toluene, and ethylacetate [121]. In the same line, Ag/SnO_2_/GO ternary nanocomposites proved relevant for acetone detection among furan, formaldehyde, chlorobenzene, and ammonia [110], while other graphene-based materials such as GO/ZnO/GO showed moderate interferences to ethanol but good selectivity among methanol and benzene [111].

One of the best selectivities to **formaldehyde** among several gases (ammonia, nitrogen dioxide, and carbon monoxide) and vapors (benzene, acetone, and methanol) all at ppb level has been identified for nonstoichiometric Co-rich ZnCo_2_O_4_ hollow nanospheres [70]. Other candidates to achieve partial selectivity to formaldehyde suggesting potential low interferences to butanol, methanol, acetone, and ammonia involve nanosheets of SnO_2_ prepared by heat treating Cu_3_SnS_4_ nanostructures [47]. NiO/MWCNTs [105] have also shown good sensitivity to formaldehyde, although the selectivity among other tested gasses, including methanol, ethanol, propanol, and acetone, was poor.

The literature also shows good responses at sub-ppm concentrations for **acetaldehyde** by employing ZnO nanostructures [39,40], however, their performance in terms of selectivity in respect to formaldehyde have proved to be poor. The information in Table 6 for ethylacetate shows excellent results for CTL sensors based on SiO_2_ and CeO_2_ nanoparticles [53] with moderate interference to acetone in the case of CeO_2_ and low interference to ether, tetrahydrofuran, ethanol, methanol, aldehydes (e.g., formaldehyde and acetaldehyde), and halogenated hydrocarbons (e.g., chlorobenzene and chloroform).

### Humidity Effects—The Uninvited Guest

The relative humidity (RH) in the atmosphere is one of the most common interfering species that has a great impact on the sensing properties of the material and its operation in a “real” environment. This is due to water molecules being sorbed at the surface (e.g., in MOXs) or in the bulk (e.g., in POMs) and abruptly changing the properties (e.g., electrical or mechanical) of the gas sensitive material. In MOXs, for instance, the adsorption of water at temperatures below 150 °C is attributed to physisorption or hydrogen bonding. However, at high temperatures (150–500 °C) the literature proposes various possible mechanisms behind the surface conductivity changes in the presence of water vapor [1]. One of these mechanisms, for instance, suggests that the OH^−^ side of water molecule can react with the Lewis acid sites (metal cation ($M_{metal}^{+}$)) forming $M_{metal}^{+}$−OH^−^, while the dissociated hydrogen from water can react with the lattice oxygen from the Lewis base or with oxygen adsorbed on surface. This mechanism involves the release of electrons (e^−^). As a result, the changes of conductivity (e.g., in resistive type sensors) or mass (e.g., in QCM type sensors) produced by the target analytes are hindered and the whole performance of the sensor is affected [71]. The possibility for hydrogen bonding interactions with water molecules at room temperature is also presented in the case of POMs, due to the polar groups on their backbone (e.g., N^δ-^–H^δ+^ in PPy and PANI). This can further result in sorption of the water molecules within the POM, which fill the free volume fraction in the polymer (e.g., swelling) and reduce the gas permeability [108]. The adsorption and permeation of water molecules within the CbMs such as graphene oxide also respond to the hydrogen bonding between water and hydrophilic groups on graphene oxide (e.g., hydroxyl groups), which can cause direct interference with gas sensing [125].

Usually, not all the reports in the literature related to VOCs sensitive materials analyze the effects of humidity ambient on the sensing performance. In fact, only a few reports of those summarized in Table 5 and Table 6 show humidity interference tests. From these results, specifically, the analysis of Co-rich ZnCo_2_O_4_ hollow spheres in 80% RH ambient shows that the response of this material to 100 ppb of formaldehyde is relatively stable with a loss of response of only 28% compared to the response registered in dry ambient [70]. Similar studies for ZnO modified with low (5%) amounts of Au to various VOCs (isoprene, ethylacetate, heptane, chlorobenzene, benzene, and formaldehyde) reported minor fluctuations of the response for RH below 35% [65]. The effect of humidity on the sensing performance of hierarchical 3D TiO_2_ nanoflowers to acetone was also tested. These studies showed that the response to acetone in 75% RH deviates only ± 4.5% from that in dry air [51].

Tests of unmodified SnO_2_ and rGO modified SnO_2_ to ethanol in a humid atmosphere (from 24 to 98% RH) registered a loss of response for both materials, although the losses were superior for SnO_2_ than for rGO-SnO_2_ [112]. Evaluation of FeO-MWCNTs and oxygen-treated MWCNTs in moisture also revealed a decrement of the response for both materials. This drop of response turned out to be larger when the humidity changed from 0 to 20% RH than when the humidity increased further from 20 to 50 or 80% RH. Moreover, the loss of response to the step changes of moisture proved to be greater for the oxygen treated MWCNTs than for the FeO-MWCNTs based sensors [104].

In the literature, one can also find different strategies to avoid the high impact of humidity in VOCs sensitive materials. The most common solutions encompass the use of external components in the sensor device as filters or dehydration elements [126]. Other strategies to reduce the impact of humidity typically include the incorporation of intentional humidity-insensitive additives (surface decoration), such as NiO [127,128], CuO [129,130], or SiO_2_ [131]. Previously, it has also been proven that the application of high-humidity (90% RH) aging treatment in MOXs such as WO_3_ [132] and SnO_2_ (the last loaded with Pd and Au) [133] significantly reduces the humidity effects at the surface while sensing toluene or a specific group of total VOCs in a range of 25 and 75% RH. Similar treatment was performed for Pt-loaded SnO_2_ films, although the humidity interference towards total VOCs for this system was found ineffective [133].

Recent studies also indicate the possibility to attenuate the humidity interference in MOXs, such as ZnO, by tuning their morphology, structural, and surface properties to increase the hydrophobicity of the surface not only at the structural level but also at the chemical level [134]. These findings are linked with those reported on oxygen treated MWCNTs, as the hydrophilic MWCNTs surface stimulated by oxygen plasma treatment made the MWCNT more sensitive to humidity compared to non-oxygen treated FeO-MWCNTs [104]. Similarly, the tests of MWCNTs/Au and MWCNTs/Au/MHDA in a humid ambient suggested that the hydrophilic nature of the carboxyl terminal from the MHDA molecules grafted to the MWCNTs/Au binds the water molecule keeping it far away from the MWCNTs, thus, providing more resilience to the MWCNTs to humidity as compared to the unmodified MWCNT [55].

## 5. Enabling the Material Properties for Their Practical Use

### 5.1. Transducing Platforms—On the Capture of the Sensor Response

**Figure 5:**
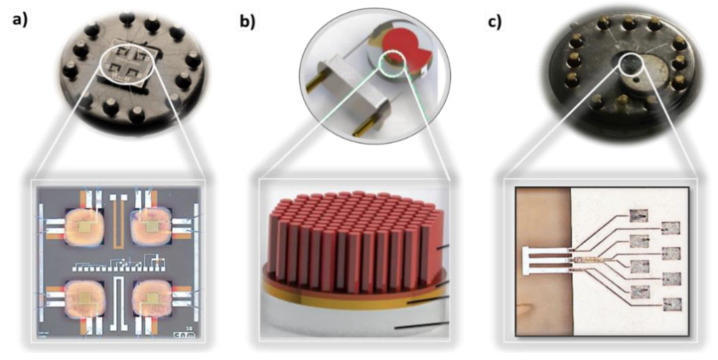
Schematic illustration of transducing platforms: (**a**) micromachined resistive, (**b**) quartz crystal microbalance, and (**c**) micromachined cantilever. Adapted from [71,141], with permission from Elsevier B.V., 2018 and 2015, respectively.

The sections above focused on the materials with the ability to sense VOCs and their main sensing properties. In practice, however, the enabling of these materials for their use in advanced applications demands their coupling with appropriate transducing platforms, which allow measuring the electrical or optical changes induced by the physicochemical phenomena experienced in the material during the gas-solid interactions. These platforms can be based on different working principles: mass-sensitive (e.g., resonating cantilevers), thermal (e.g., pellistors, thermoelectric, or Seebeck-effect-based sensors), optical (e.g., phosphorescence/fluorescence or chemiluminescence sensors), or electrochemical (e.g., conductometric and potentiometric sensors) [135]. Figure 5 displays examples of transducing platforms for measuring resistive and mass-load changes. Electrochemical-based transducing platforms are the most common approach in the literature and commercial field, probably due to their relatively simple fabrication process and characterization. Within this group, chemoresistive sensors are generally the most representative and this fact is reflected in Table 8 and Table 9, in which most of the accounted materials for VOCs report the operation under the resistive principle. One can see in these tables that VOCs sensitive materials are also enabled by monitoring optical parameters such as CTL [53,102,119] or LSPR [66,67], and mass-load changes mainly using QCM [71], although with less frequency. From this, it is concluded that the properties of VOCs sensitive materials can be exploited using various physical changes so the systems are not limited to only resistive changes. Enabling the signal capture of the different physical changes, separately or in conjunction, would allow for VOCs sensing systems with better selectivity.

Back in the past, most of the technology for gas sensing transducing platforms relied on thick film technology, particularly using silica or alumina substrates. Currently, however, the transducing platforms exploit micro/nano fabrication technologies (usually based on silicon as substrate), which makes it possible for their incorporation into integrated circuits (IC) at micro/nano scale in a single chip [135,136]. During the past decades, various types of gas/vapor sensors (resistive, thermal, mass-sensitive, optical) have been reported in the literature using standard micro-electro-mechanical systems (MEMS) technology. This technology for instance has been shown to facilitate the formation of thermally well-isolated structures for more efficient conductometric and Seebeck-effect based sensors or the formation of miniaturized cantilevers for mass-sensitive based sensing. Recently, miniaturized light platforms for the photoactivation instead of thermoactivation of gas sensitive material have also been developed [137].

Another significant advantage of using micro/nano fabrication technologies is the possibility to integrate several different transducers on a single chip along with the driving and signal conditioning circuitry or other smart features (e.g., wireless communication) to build electronic noses with potentially low cost via mass production [135]. A good approximation of this concept, in which various transducing principles were implemented monolithically in a microsystem to operate simultaneously, was developed previously using micro/nano fabrication technology [138]. Recently, other emerging technologies based on flexible substrate materials such as polymers, textiles, and paper are positioning well in electronics and thus in sensing systems (including VOCs sensors) [139,140]. These novel technologies can introduce biocompatibility, reusability, and/or biodegradability to consumer electronics, alleviating environmental issues and reducing significantly the costs associated with recycling operations.

Therefore, micro/nano technologies have the potential to contribute and enable improving further the properties of materials for different applications. VOCs monitoring systems built by micro/nano technologies may have an impact in various fields and cover the current demands of traditional markets focused on the industry and environmental control, as well as emerging niches dedicated to the food industry, agriculture, and medicine. Most of these fields are keen to implement solutions in line with the digital future and the smart anything everywhere (SAE) concept, in which VOCs sensors could be the next family of sensors for smart portable devices. Hence, there is a need to go further in the effective integration and synthesis of sensitive materials into miniaturized systems with low power consumption and reduced fabrication costs at a large-scale.

### 5.2. Synthesis of Materials—Path to the On-Chip Integration

The integration of transducing platforms and material synthesis methods is an essential phase for VOC sensors’ scalability and large production. Such integration can mostly be achieved by direct or transfer methods [140,142]. The first (direct method) involves the selective deposition of the material over the transducing platform. Direct methods can reduce the processing time and steps of the whole sensor assembling process, particularly when using bottom-up approaches (i.e., synthesis of materials through assembling of atoms derived from chemical precursors) rather than top-down approaches (i.e., based on carving, slicing, or etching a macroscale material source). The second (transfer method) relies on the use of pre-synthesized materials and their dry or wet transfer (re-deposition) over the transducing platform. Transfer methods can facilitate a broad choice of materials and modifications and can be especially useful when integrating aligned single 1D structures into a transducing platform, although one must be aware of the surface contamination issues which entail most of the transfer procedures [142]. Dielectrophoresis [143] and contact printing using polydimethylsiloxane (PDMS) [144] are two examples of the wet and dry transfer approach, respectively.

Transfer methods are by far the most used approaches in the literature for integrating the materials into the transducing platforms. Table 8 and Table 9 evidence this fact, indicating a preference for developing VOCs sensitive materials by wet chemical synthesis (WCS) and their subsequent transfer (or re-deposit) over the transducing platforms. The preferred WCS methods for VOCs sensitive MOXs, POMs, or CbMS include sonochemical process [39,40], hydrothermal process [36,46,47,50,51,52,73,102], one-pot wet-chemical method [37], photodeposition [65], sol-gel method [74], microwave-assisted approach [45,112,116], precipitation [69,70,105,122], calcination of precursors [119], seed mediated growth method [66], pyrolysis [121], spray deposition [116], Langmuir–Blodgett method [123], chemical bath deposition [111], electrochemical methods, and chemical oxidation polymerization [58]. The transfer methods are generally based on the formation of pastes or suspended solutions using common solvents (e.g., ethanol, terpineol) for their subsequent printing or drop coating. Previously, it was found that the synthesis and the post-treatment process of materials (e.g., MWCNTs) can also facilitate the transfer procedures by improving the solubility or dispersion of materials [104].

The reports centered on WCS synthesis concentrate regularly on tuning conditions and finding their correlation with the material functionality towards common VOCs. An example of the synthesis of ZnO shows that different salts (e.g., zinc acetate, zinc chloride, or zinc sulfate) in hydrothermal synthesis influence the morphologies and microstructural features of the porous ZnO products. Measurements on the gas sensing properties of the porous products to VOCs (e.g., acetone, chlorophenol, and formaldehyde) revealed improved sensing characteristics for porous ZnO synthesized using zinc sulfate compared to those obtained using zinc chloride and zinc acetate. A similar example on the synthesis of SnO_2_ shows that the use of a starch template for the hydrothermal synthesis of worm-like nanostructured SnO_2_ and the variation of starch content has a great impact on the microstructure of the final product. The gas-sensing tests of this study exhibited better sensing properties towards butanol for the material processed with higher content (2 g) of starch [46]. In the same way, the sensor fabricated with 0.05 wt.% of MWCNTs showed to be selective to ethanol and methanol while the sensor with 0.1 wt.% of MWCNTs demonstrated selectivity to acetone and ether among the other tested VOCs [106]. These examples and other similar ones in the literature often attribute the improvements to the differences in morphology and/or surface area [36]. The effect of the impurity levels derived from the synthesis and transfer-processing steps are generally less discussed, despite there being evidence that residual ions (e.g., chlorine, sulfur, alkali and alkaline earth metals) account for the widespread of physical and chemical properties of similar oxide materials [145].

Direct methods for integrating VOCs sensitive materials into transducing platforms are a minority in Table 8 and Table 9. Among the representative techniques for direct integration are sputtering [38], chemical vapor deposition (CVD) [48,49], and hydrothermal [51] and electrochemical deposition [71]. These techniques allow for the selective integration of films and structures by implementing masks or patterns on desired locations. Even though these techniques are not exempt from introducing residual impurities during material processing, the in-situ integration approach with no further manipulation or extra steps diminishes considerably the contamination of the material surface. An example of a direct method using CVD shows that the integration of one-dimensional WO_3_ structures modified with Au or Pt either over MEMS- or polymer-based transducing platforms is feasible in a single step. These structures reported enhanced sensing properties to acetone or methanol as a function of the surface modification with Au or Pt, respectively. Another example of the use of the direct method shows the integration by electrodeposition of ZnO rods modified with Pd over QCM-based transducing platforms [71]. As for the previous example, this report also emphasized the improvement of sensing properties to ethanol and xylene by modifying the ZnO structures with Pd. Overall, the literature search suggests that there is still a lot of room for investigating synthesis methods for direct integrations of tuned VOCs sensitive materials.

### 5.3. Machine-Learning—Mimicking the Human Olfactory Systems

**Figure 6:**
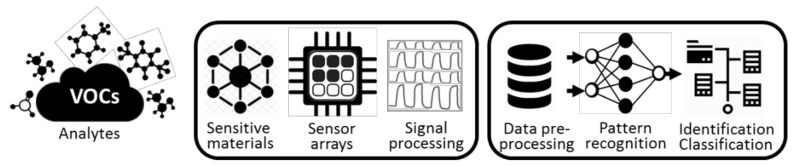
Block representation of the main constituents of an e-nose system. The hardware block includes the sensitive materials, transducers, integrated sensor arrays, and signal processing elements. The software or machine learning block includes the data collection and preprocessing, featuring extraction, pattern recognition, and classification algorithms.

Another essential phase in the practical use of gas/vapor sensitive nanomaterials and systems is their coupling with appropriate machine learning algorithms. The ensemble of these elements, i.e., hardware and software, is usually known as electronic nose (e-nose) technology, in reference to their approximation to reproduce the human olfactory system [146,147]. In e-noses, the selection of suitable materials and transducers for the sensor array is accompanied by the selection of proper machine-learning algorithms to comprehend the large amount of data delivered by the sensor array and identify/classify the information with the aim to make the system more selective and precise for practical implementations. Figure 6 displays a summarized representation of the main constituents of an e-nose system. The figure shows the hardware stage (i.e., materials, transducers, and sensor arrays) and the machine-learning stage, which involves the data collection, modeling, training, and evaluation steps. So far, this review has focused on the hardware stage, and in particular on VOC sensitive materials, whereas this section aims to provide the reader with a complementary overview of the basic attributes of e-noses and their application. As the scope of this topic is vast, the section is not intended to be exhaustive and for deeper insight into e-noses, their principles, and application, we invite the reader to revise recent reviews, for instance, [148,149,150,151,152].

Amongst a host of machine-learning algorithms, those based generically on pattern recognition methods, i.e., algorithms that recognize (ir)regularities or specific patterns in the data to subsequently classify them by means of training models, are frequent in e-nose applications. In this context, methods such as principal component analysis (PCA), discriminant analysis (linear—LDA, function—DFA, stepwise—SDA), regressions (partial least squares—PLSR, generalized least squares—GLSR, multiple linear—MLR), support vector machines (SVMs), and artificial neural networks (ANN) are the most sounded data analysis methods [148].

Typical e-nose applications, in which VOC analytes are potentially involved, include the discrimination of samples (not necessarily specific analytes) by finding differences in the patterns to identify, for instance, the meat origin [153], rice aging [154], beverage brands [155], coffee beans [156], or controlled populations of healthy and unhealthy subjects [157,158,159]. However, there are also other studies, which target the discrimination of more specific analytes and their concentrations. Table 10 summarizes a few examples in this context with focus on e-noses with (nano)materials-based sensor arrays. In terms of algorithmic tools, the works make references especially to PCA, LDA, and ANN methods [160,161,162,163,164]. Whilst PCA and LDA methods are useful tools for reducing the dimensionality of measurement space and extracting the information for pattern recognition, ANN methods are usually employed for classification tasks, although ANN can also be trained to learn sample patterns and predict responses for unidentified samples. The use of deep learning tools with basis on ANN methods to automatically detect and classify information in raw datasets (without pre-processing) are also relatively new alternatives in e-noses [148,155]. As noticed in Table 10, the sensor elements used in e-noses seems to be characterized specially for the use of resistive sensors based on MOXs. The justification for this is frequently supported by the broad gas/vapor sensitivity of these components, their low cost, and the need of simple drive circuits, amongst other characteristics such as fast and stable responses and long life [146,157,161,165,166].

Certainly, the application of machine learning for artificial olfactory systems has demonstrated further solving the selectivity issues of (nano)material-based gas/vapor sensors. In fact, the reports on e-noses show that partial selective commercial sensors can provide sufficient data to discriminate and classify samples [161,162,163,165,166,167,169]. However, despite e-noses technology apparently being resolved by the existing sensors, there is still a continuous demand in the field for more miniaturized sensor components with broader gas/vapor sensitivity, lower detection limits (at ppb-ppt level), low power consumption, low cost, and operation without consumables; features which are still not fully available in current commercial sensors [172]. These new characteristics would allow for even more generalized use of e-noses in the fields in which they have already shown their potential. Hence, new developments in materials and system integration as the primary components of the value chain are still timely to pave the way towards advanced artificial olfactory systems.

## 6. Outlook and Conclusions

Nanomaterials based on metal oxides, conductive polymers, and carbon-based materials are representative in VOCs sensing. Their optimization and enhanced properties generally involve the control of morphology, size, and composition. The most successful systems include structured morphologies, particularly hierarchical structures at the nanometric scale with intentionally introduced tunable “decorative impurities” or well-defined interfaces forming bilayer structures. These groups of modified or functionalized structures, in which metal oxides are still the main protagonists either as host or guest elements, have proved improvements in VOCs sensing. Whilst the main improvements usually include better and faster responses, lower operating temperatures, or better stability, the advances in terms of selectivity are still blurred. First, because most reports in the literature concentrate on studying a reduced number of VOCs, among them especially simple oxygenated hydrocarbons, and second because the selectivity is evaluated by the response comparison of single analytes often with incomparable concentrations among them. Overall, the literature survey points out the need for investigating more in depth the material functionality to larger groups of VOCs as well as the convenience of probing other transducing principles rather than resistive for nanomaterial-based sensors. In terms of materials, the needs are directed overall to explore new hybrid material combinations that involve the formation of nanoscale interfaces between (i) inorganic and organic semiconductors (e.g., MOXs and POMs), (ii) inorganic and carbon-based materials (MOXs and graphene), and (iii) organic and carbon-based materials (e.g., POMs and graphene) to exploit the synergistic effects of these combinations. It is worth mentioning the availability of a broad group of emerging materials with less tradition in gas sensing, but with promising results to operate alone or in combination with traditional gas-sensitive materials. Within this group, the exploration of (i) perovskite and spinel oxides with multications variations; (ii) transition metal dichalcogenides (TMDs) and transition metal carbides/nitrides (MXenes); and (iii) nitrides, and phosphorene, silicene, or germanene are attractive. For both groups (i.e., traditional and emerging gas-sensitive materials), the new research efforts need to be invested in (i) controlling the load of constituents and their correlation with the VOC sensing patterns (information rarely found in the reports), (ii) finding dedicated materials for detecting compounds from the group of aliphatic hydrocarbons and carbonyl compounds, (iii) exploring the possible multifunctional properties in the material for exploiting mixed output concepts (e.g., electric, optic, mechanic), and (iv) developing synthetic methods for a more realistic use of the materials in miniaturized integrated systems and their projection into the application market. The solution to those needs will pave the way to the selective detection of specific VOCs in the future and the generalized use of artificial olfactory systems adapted to the society demands.

## Figures and Tables

**Table 1:** Summary of the volatile organic compounds (VOCs) profiles in air-quality due to anthropogenic emissions in the atmosphere [24] and exhaled breath due to cancer (e.g., lung, breast, gastroesophageal, colorectal, oral cavity, head and neck, etc.) [21]. The presented threshold limit values (TLV) are based on the documentation of the American Conference of Governmental Industrial Hygienists (ACGIH) from 2019 [25]. The concentration range in the exhaled breath (CREB) for the selected gases/vapors, which include the minimum and maximum levels found within healthy and unhealthy subjects, were adopted from the references [26,27,28,29,30]; these references are also summarized in reference [21].

VOCs Monitoring							
Air Quality				Breath Analysis			
Anthropogenic VOCs emission	**VOCs** **Profile**	**Representative** **Vapor Analytes**	**TLV (ppm)**	Cancer	**VOCs Profile**	**Representative** **Vapor Analytes**	**CREB (ppb)**
	Alcohols	Methanol Ethanol 1-Propanol	200 ^TWA^ 1000 ^TWA^ 200 ^TWA^		Alcohols	Methanol Ethanol 1-Propanol	157–344 96–2848 4–13
	Ethers	Furan	N/A				
	Aldehydes	Formaldehyde Acetaldehyde Propenal	0.1 ^TWA^ 25 ^C^ 0.1 ^C^		Aldehydes	Pentanal Heptanal Nonanal	2–7 2–7 2–107
	Ketones	Acetone 2-Butanone	250 ^TWA^ 200 ^TWA^		Ketones	Acetone 2-Butanone 3-Hydroxy-2-Butanone	35–1000 0.002–3 0.002–0.05
	Esters	Ethylacetate	400 ^TWA^				
	Carboxylic acid	Acetic acid	10 ^TWA^				
	Alkanes	Ethane Propane Butane Pentane Hexane Heptane Octane Nonane Decane Undecane Dodecane	N/A N/A 1000 ^TWA^ 1000 ^TWA^ 50 ^TWA^ 400 ^TWA^ 300 ^TWA^ 200 ^TWA^ N/A N/A N/A		Alkanes	Pentane 4-Methyloctane	2–18 16–19
	Alkenes	Ethylene Propylene Butylene 1,3-Butadiene	200 ^TWA^ 500 ^TWA^ 250 ^TWA^ 2 ^TWA^		Alkenes	Isoprene	41–109
	Alkynes	Acetylene	N/A				
	Aromatics	Benzene Ethylbenzene Styrene Toluene Xylene	0.5 ^TWA^ 20 ^TWA^ 10^TWA^ 20 ^TWA^ 100 ^TWA^		Aromatics	Ethylbenzene Toluene	1–18 1–37
	Halohydrocarbons	Chloroform Dichloromethane Dichloroethane Chlorobenzene	10 ^TWA^ 50 ^TWA^ 100 ^TWA^ 10 ^TWA^				
	Other	Acetonitrile Dimethyl disulfide Limonene	20 ^TWA^ 0.5 ^TWA^N/A				

8-h time weighted averages (TWA)—the average values of exposure over the course of an 8-h work shift; ceiling values (C)—the exposure limits that should not be exceeded at any time; N/A—data not available.

**Table 2:** Comparative table showing the main properties of MOXs, POMs, and CbMs. The symbols attached to each qualitative estimation remark the degree, major (↑) or minor (↓), of each characteristic with respect to the other groups of materials.

Properties	MOXs	POMs	CbMs	Notes
Sensitivity	High↑↑	High↑	High	Advantageous for all materials to specific gases/vapors, although in major degree for MOXs with respect to POMs and CbMs.
Selectivity	Poor↓	Poor	Poor	Poor for all materials within a large number of gases/vapors. POMs and CbMs have shown advantages for specific gases such as NH_3_ and NO_2_.
Stability	High	Medium↓	Medium	More advantageous for MOXs due to operation at high temperatures. The stability of POMs is highly dependent on humidity.
Speed of response	High	Low	Low	More advantageous for MOXs due to operation at high temperatures.
Long-lasting lifetime	High	Low↓	Low	More advantageous for MOXs due to low probability of poisoning and degradation. POMs have more probability of degradations in humid ambient with respect to CbMs.
Operating temperature	High	Low	Low	More advantageous for POMs and CbMs due to the capacity of adsorption at room temperature.
Energy consumption	High	Low	Low	More advantageous for POMs and CbMs as they do not require thermal activation.
Cost	Low↓	Low	High	More advantageous for MOXs and POMs due to the wider choice of synthesis methods.
Miniaturization potential	High↑	High	High	Advantageous for all materials according to the transducing principle. MOXs are more advantageous in this area due to their proved compatibility with micro/nano fabrication processes, although the recent scalable integration methods based on roll-to-roll and printed electronics are facilitating more the scalable integration of POMs and CbMs into miniaturized systems.

**Table 3:** Summary of VOCs sensitive materials reported in the literature according to their dimensionality and/or morphology.

Materials	Morphology	Features	Ref.
0D	Particles	Diameters of 1–100 nm	[45,48,49,52,65,66,67,68]
	Cubes	Average size of 200 nm	[69]
	Hollow spheres	Diameter of 72 nm	[70]
1D	Rods and needles	Diameters of 100–200 nm and lengths up to 10,000 nm	[48,49,52,71]
	Rings	Diameters of 120 nm (inner) and 180 nm (outer) and lengths of 1000–1500 nm	[40]
2D	Sheets and monolayers	Size of 100–400 nm or as large as 30–70 µm and thickness of 10–60 nm	[57,72]
3D	Porous films	Pore size of 6–150 nm	[36,38,45,46,73]
	Flowers	Sheet size of 120–2000 nm and thickness of 12–50 nm	[37,39,47,50,51,74,75]
	Tetrapods	Rod diameters of 50 nm and lengths of 1000 nm	[76]

**Table 4:** Sensing properties of pristine gas sensitive materials to VOCs.

Material	Morphology	Size, nm	VOCs	c, ppm	T, °C	R	t_R_, s	t_r_, s	Ref.
SnO_2_	NPs	9.1 ^CS^	Butanol	10	150	190	9	N/A	[46]
	NPs	6.1 ^CS^			150	630	11	N/A	
SnO_2_	NPs	6.3 ^CS^	Toluene	100	260	9.7	3	10	[47]
	NPs ^com^	N/A			240	6.2	N/A	N/A	
ZnO	NPs ^com^	10–100 ^Ø^	Ethanol	100	N/A	2.6	16	100	[37]
	NShs	1000 ^T^			N/A	8.5	10	80	
ZnO	NTPs (NRds)	50 ^Ø^ 1000 ^L^	Ethanol	500	340	57	50	70	[76]
ZnO	NPs	N/A	Acetaldehyde	0.05	220	2	N/A	N/A	[40]
	NRgs	N/A			220	1.1	N/A	N/A	
TiO_2_	NPs	10 ^Ø^	Acetone	200	400	7.8	240	N/A	[77]
	NShs	120 ^L^ 12–23 ^T^		700	60	1.7	10	45	[51]
CuO	NShs	50 ^T^	Ethanol	0.2	250	2.1	11.2	8.4	[74]
Co_3_O_4_	cNCs	2000 ^S^ 50 ^T^	Ethanol	10	300	1.7	<10	<10	[69]
	ML	17.6 ^CS^		5	200	114	N/A	N/A	[72]
In_2_O_3_	NShs-hMSp	20 ^T^	Acetone	5	350	1.8	4	10	[50]
	NShs-MSp	20 ^T^			350	1.7	4	10	
Co_3_O_4_	HSps		Formaldehyde	1	225	1.3	N/A	N/A	[70]
ZnCo_2_O_4_	HSps	72 ^Ø^			225	7.3	149	497	
SWCNTs	SWCNTs	6 ^Ø^ 2000 ^L^	Toluene	N/A	RT	7.5 *	N/A	N/A	[85]
MWCNTs	MWCNTs	20 ^Ø^ 4000 ^L^			RT	2.0 *	N/A	N/A	

NPs: nanoparticles, ^com^: commercial powder, NShs: nanosheets, NTP: nanotetrapods, NRds: nanorods, NRgs: nanorings, cNCs: concave nanocubes, ML: monolayer, hMSp: hierarchical microsphere, MSp: microsphere, HSps: hollow spheres, SWCNTs: single-walled carbon nanotubes, MWCNTs: multi-walled carbon nanotubes, ^CS^: crystal size, N/A: data not available, ^Ø^: diameter, ^T^: thickness, ^L^: length, ^S^: size, RT: room temperature, c: concentration, T: operating temperature, R: response defined as R_a_/R_g_, t_R_: response time, t_r_: recovery time, and * sensitivity (kHz/ppm).

**Table 5:** Sensing properties of modified and unmodified gas sensitive materials to VOCs.

Material	Loading	VOCs	c, ppm	T, °C	R	t_R_, s	t_r_, s	Ref.
Au@ZnO	Au 3 at.%	Isoprene	50	360	31	N/A	N/A	[65]
ZnO	Au 0 at.%				25	N/A	N/A	
Au@ZnO	Au 5 at.%	Methanol	50	360	21	4	3	[65]
ZnO	Au 0 at.%				4.8	N/A	N/A	
Ce-doped SnO_2_	Ce 2 at.%	Formaldehyde	500	320	10	8	4	[73]
SnO_2_	Ce 0 at.%				28	10	5	
Mg-doped SnO_2_	Mg 1 at.%	Ethanol	80	160	14	143	N/A	[103]
SnO_2_	Mg 0 at.%				1	N/A	N/A	
Pd@TiO_2_	Pd 12 at.%	Isopropanol	5000	200	4.4	N/A	N/A	[52]
TiO_2_	Pd 0 at.%				1.6	N/A	N/A	
Fe_2_O_3_@WO_3_	Fe 3.9 at.%	Toluene	100	220	8	150	15	[49]
Pt@WO_3_	Pt 3.7 at.%				7.8	100	30	
WO_3_	Fe & Pt 0 at.%				2.5	400	170	
CuO/SnO_2_	CuO 3 mol%	Xylene	50	280	10	N/A	N/A	[45]
SnO_2_	CuO 0 mol%				2.5	N/A	N/A	
ZnO-CuO	Cu 65 at.% Zn 35 at.%	Acetone	10	300	1.2	22	26	[75]
TiO_2_/SnO_2_	N/A	Benzene	100	220	13,000 *	N/A	N/A	[102]
SnO_2_	N/A				5000 *	N/A	N/A	
Pt@MWCNTs	Pt 1.8 at.%	Methanol	11	RT	1.01	N/A	N/A	[54]
Pd@MWCNTs	Pd 2.1 at.%				1.01	N/A	N/A	
MWCNTs	O plasma treat.				1.01	N/A	N/A	
FeO-MWCNTs	Fe 2 at.%	Toluene	5.8	RT	1.02	489	N/A	[104]
MWCNTs	O plasma treat.				1.07	457	N/A	
NiO/MWCNTs	N/A	Ethanol	500	180	3	27 ^100^	87 ^100^	[105]
NiO	N/A				1.5	N/A	N/A	
ZnO/MWCNTs	0.1 wt.% CNTs	Acetone	320	270	62	41	90	[106]
ZnO	0 wt.% CNTs				7	79	108	
Ag/PPy	1:10 v.r.	Acetone	5	RT	5 ^kHz^	150	230	[107]
PPy	N/A				3 ^kHz^	160	240	
CdTe/PPy	0.03 at.% Cd	Acetone	5	RT	4 ^kHz^	155	270	[108]
PPy	N/A				3.5 ^kHz^	165	290	
Ti-PTFE	N/A	Acetone	2000	RT	1.03	N/A	N/A	[109]
PEDOT-PSS/GO	GO 0.04 wt.%	Methanol	35	RT	11	3.2	16	[57]
PEDOT-PSS	GO 0 wt.%				1	N/A	N/A	
PANI PANI-CD	N/A N/A	Benzene	150	RT	45.65 ^NCC%^ 57.66 ^NCC%^	N/A	N/A	[68]
PANI PANI-CD	N/A N/A	Toluene	46	RT	39.36 ^NCC%^ 40.68 ^NCC%^	N/A	N/A	[68]
Ag/SnO_2_/GO	N/A	Acetone	150	300	1.26	2	8	[110]
SnO_2_/GO	N/A				1.05	3	50	
GO/ZnO	N/A	Acetone	200	450	7	<14	N/A	[111]
GO/ZnO/GO	N/A				7	<12	N/A	
ZnO	N/A				12	<13	N/A	
rGO-SnO_2_	23.7 at.% C, 38.4 at.% Sn, 37.9 at.% O	Ethanol	100	300	70	11	N/A	[112]
SnO_2_	14.6 at.% C, 45.2 at.% Sn, 40.2 at.% O		100	300	62	9	N/A	

MWCNTs: multi-walled carbon nanotubes, PPy: polypyrrole, PTFE: polytetrafluoroethylene, PEDOT: poly(3,4-ethylenedioxythiophene), PSS: poly(styrenesulfonate), PANI: polyaniline, CD: cyclodextrin, GO: graphene oxide, rGO: reduced graphene oxide, v.r.: volumetric ratio, N/A: data not available, * CTL sensors: the response represents the relative CTL intensity, ^100^: response and recovery time for 100 ppm, ^kHz^: SAW sensors: frequency shift, ^NCC%^: sensor efficiencies in%, RT: room temperature, c: concentration, T: operating temperature, R: response defined as R_a_/R_g_, t_R_: response time, and tr: recovery time.

**Table 6:** Cross-responses to different VOCs recorded using MOXs. The color codes and numbers in each column of the table represent the load of response for the referenced material towards the same gas concentration of various tested analytes. N/A: data not available, RT: room temperature.

	400 °C—100 ppm	220 °C—1 ppm	220 °C—500 ppb	220 °C—100 ppm	250 °C—87 ppm	340 °C—500 ppm	360 °C—50 ppm	RT—N/A	150 °C—10 ppm	280 °C—50 ppm	260 °C—100 ppm	320 °C—500 ppm	220 °C—2000 ppm	220 °C—2000 ppm	220 °C—2000 ppm	220 °C—100 ppm	60 °C—700 ppm	200 °C—5000 ppm	350 °C—50 ppm	250 °C—200 ppb	300 °C—10 ppm	250 °C—5 ppm	210 °C—2000 ppm	210 °C—2000 ppm	210 °C—2000 ppm	210 °C—2000 ppm	210 °C—800 ppm	220 °C—100 ppm	280 °C—50 ppm	300 °C—10 ppm	210 °C—2000 ppm	225 °C—400 ppb	N/A—N/A	RT—3800 ppm
Methanol	**❿**					**❸**	**❻**	**❷**	**⓿**	**❻**	**❺**	**❽**	**⓿**	**❸**	**❿**		**❶**	**❶**					**⓿**	**⓿**	**❹**	**❸**	**⓿**	**⓿**	**❻**		**❷**		**❿**	
Ethanol				**❿**	**❿**	**❿**		**❽**	**❶**	**❿**	**❼**	**❽**	**❶**	**❺**	**❼**	**❼**		**❷**		**❿**	**❿**	**❹**	**❶**	**⓿**	**❸**	**❹**			**❼**	**❻**	**❶**	**❷**	**❼**	
Chloroethanol	**❻**																																	
Propanol					**❺**			**❹**									**❶**	**⓿**									**⓿**	**⓿**					**❺**	
Butanol									**❿**		**❸**																**❶**							
Pentanol																																		**❿**
Ether																							**❹**	**❸**	**❽**	**❿**					**❿**			
Diethylether							**❾**																					**❶**						
Tetrahydrofuran																							**❸**	**❹**	**❸**	**❹**					**❸**			
Methane																			**❷**															
Heptane							**❶**																											
Octane																																		**❶**
Decane							**❸**																											
Isoprene							**❿**																											
Benzene							**❷**		**⓿**	**❶**	**❽**										**❶**	**❶**	**⓿**	**⓿**	**❷**	**⓿**	**❿**	**❿**	**❻**		**❶**	**❶**		
Ethylbenzene										**❷**																			**❽**					
Chlorobenzene	**❶**						**❼**																**❷**	**⓿**	**⓿**	**❷**					**❷**			**❼**
Toluene				**❸**	**❸**		**❶**	**❸**	**⓿**	**❶**	**❿**		**❹**	**❺**	**❷**	**❿**	**⓿**		**❾**		**❷**	**❺**	**⓿**	**⓿**	**❷**		**⓿**	**❹**	**❽**		**⓿**			**❷**
Xylene							**❶**	**❿**		**❷**										**❼**		**❿**					**⓿**	**⓿**	**❿**					**❿**
Chlorophenol	**❾**																																	
Acetone	**❽**			**❹**	**❻**	**❸**	**❷**	**❶**	**⓿**	**❿**	**❻**	**❿**	**❿**	**❿**	**❷**		**❿**		**❿**	**❾**	**❹**		**❸**	**❼**	**❿**	**❺**			**❼**	**❿**	**❸**	**❶**		
Butanone																	**❸**										**⓿**	**⓿**						
Formaldehyde	**❸**	**❿**	**❺**			**❷**	**❸**		**❷**	**❶**	**❾**	**❻**							**❶**	**❾**		**❶**	**⓿**	**⓿**	**❶**	**⓿**	**⓿**	**⓿**	**❷**		**❶**	**❿**		
Acetaldehyde		**❿**	**❿**																				**⓿**	**⓿**	**❶**	**❷**		**⓿**			**❶**			
Hexaldehyde							**❼**																											
Ethylacetate							**❽**	**❶**															**❿**	**❿**	**❸**	**❸**					**❷**			
Butylacetate							**❺**																											
Chloroform								**❶**											**❻**				**❶**	**⓿**	**❼**	**❶**					**❶**			
Trichloroethylene							**❷**																											
NH_3_										**❻**	**❸**																	**⓿**	**❹**	**❻**		**⓿**		
H_2_																**❺**		**❿**																
NO_2_																														**❸**		**❶**		
CO																						**⓿**										**⓿**		
H_2_S																														**❺**				
	ZnO [36]	ZnO [39]	ZnO [40]	ZnO [37]	ZnO [38]	ZnO [76]	Au@ZnO [65]	Pd@ZnO [71]	SnO_2_ [46]	SnO_2_ [45]	SnO_2_ [47]	Ce-dop. SnO_2_ [73]	WO_3_ [48]	Au@WO_3_ [48]	Pt@WO_3_ [48]	Fe_2_O_3_@WO_3_ [49]	TiO_2_ [51]	Pd@TiO_2_ [52]	In_2_O_3_ [50]	CuO [74]	Co_3_O_4_ [69]	Co_3_O_4_ [72]	SiO_2_ [53]	CeO_2_ [53]	Y_2_O_3_ [53]	La_2_O_3_ [53]	Au/La_2_O_3_ [119]	TiO_2_/SnO_2_ [102]	CuO/SnO_2_ [45]	ZnO-CuO [75]	SiO_2_/Fe_3_O_4_ [53]	ZnCo_2_O_4_ [70]	Au [66]	Ag [67]
Response load in%		0			10			20			30			40			50			60			70			80			90			100		
Number		⓿			❶			❷			❸			❹			❺			❻			❼			❽			❾			❿		

**Table 7:** Cross-responses to different VOCs recorded using POMs and CbMs. The color codes and numbers in each column of the table represent the load of response for the referenced material towards the same gas concentration of various tested analytes. N/A: data not available, RT: room temperature.

	RT—10 ppm	RT—10 ppm	RT—N/A	250 °C—320 ppm	250 °C—320 ppm	RT—N/A	300 °C—500 ppb	RT—N/A	N/A—1000 ppm	RT—N/A	RT—5000 ppm	RT—200 ppm	300 °C—150 ppm	450 °C—200 ppm	450 °C—200 ppm	RT—20 ppm	RT—20 ppm	180 °C—100 ppm	RT—5 ppm	RT—5 ppm	RT—10 ppm	300 °C—150 ppm	RT—5 ppm	RT—5 ppm	RT—5 ppm	RT—5 ppm	RT—250 ppm
Methanol	**❿**	**❿**	**❿**	**❹**	**❿**			**❸**		**❺**		**❿**		**❾**	**❷**	**❻**	**❷**	**❾**	**❶**	**⓿**	**⓿**	**❻**					
Ethanol	**❾**	**❾**	**❽**	**❹**	**❺**	**❾**	**❺**	**❺**	**❿**	**❿**		**❸**		**❾**	**❽**	**❿**	**❿**	**❿**	**❶**	**⓿**	**⓿**	**❿**	**❾**	**❼**	**❽**	**❻**	
Propanol								**❻**		**❿**	**❹**	**❶**						**❾**									
Ether				**❸**	**❶**																						
Furan													**❷**														
Hexane						**❷**																					
Benzene			**❻**				**⓿**							**❸**	**❷**	**❽**	**⓿**		**❶**	**❷**		**❶**					**❷**
Chlorobenzene												**❶**	**❶**														
Ethylbenzene																											**❾**
Toluene			**❸**			**❿**	**❶**	**❿**		**⓿**	**❿**	**❶**							**❿**	**❿**	**⓿**	**⓿**	**❹**	**❹**	**❹**	**❸**	**❽**
Xylene																						**⓿**					**❿**
Acetone	**❸**	**❷**	**❾**	**❿**	**❷**	**❷**	**❿**	**⓿**	**❿**	**❺**		**❷**	**❿**	**❿**	**❿**	**❺**	**❸**	**❾**	**❶**	**❶**		**❷**	**❿**	**❿**	**❿**	**❿**	
4M2P											**❽**																
Formaldehyde													**❸**					**❽**			**⓿**						
Ethylacetate							**❹**	**❷**		**❺**																	
Acetic acid										**❺**																	
Acetonitrile										**❺**																	
Triethylamine										**❺**																	
NH_3_										**❿**			**⓿**								**❿**						
H_2_																									**❹**		
CO																									**❺**		
Water										**❺**	**⓿**	**❷**															
	Pt@MWCNTs [54]	Pd@MWCNTs [54]	PEDOT-PIL@Fe_3_O_4_ [116]	ZnO/MWCNTs(0.1 wt.%) [106]	ZnO/MWCNTs(0.05 wt.%) [106]	DT-capped Au [122]	MOF-ZnO [121]	SWCNTs/CdA [123]	Ti-PTFE [109]	Ag-BC-bsh [117]	Au-DT [124]	PEDOT-PSS/UL-GO [57]	Ag/SnO_2_/GO [110]	GO/ZnO [111]	GO/ZnO/GO [111]	MWCNTs/Au [55]	MWCNTs/Au/MHDA [55]	NiO/MWCNTs [105]	MWCNTs [104]	FeO/MWCNTs [104]	PPy/rGO [58]	rGO/SnO_2_ [112]	PPy [107]	PPy [108]	Ag/PPy [107]	CdTe/PPy (1:10) [108]	SWCNTs-calixarene [120]
Response load in%						0		10		20		30		40		50		60		70		80		90		100	
Number						⓿		❶		❷		❸		❹		❺		❻		❼		❽		❾		❿	

**Table 8:** Summary of MOXs for VOCs sensing, their features and corresponding synthesis method and integration.

Material	Morphology	Features, nm	Method	Integration	Principle	Ref.
ZnO	PF	27 ^PS^	WCS	Transfer	Resistive	[36]
ZnO	NShs	200–400 ^S^ 10–60 ^T^	WCS	Transfer	Resistive	[39]
ZnO	NRgs	180 ^OØ^ 120 ^IØ^ 1000–1500 ^L^	WCS	Transfer	Resistive	[40]
ZnO	NShs (NFls)	1000 ^S^	WCS	Transfer	Resistive	[37]
ZnO	PF	33.8 ^CS^	Sputtering	Direct	Resistive	[38]
ZnO	NTPs (NRds)	1000 ^L- NRds^ 9 ^Ø- NRds^	PVD	Transfer	Resistive	[76]
Au@ZnO	NPs@NShs	9 ^Ø-NPs^	WCS ^PhA^	Transfer	Resistive	[65]
Pd@ZnO	NPs@NRds	200 ^Ø-NRds^	WCS	Direct	QCM	[71]
SnO_2_	PF	6–15 ^Ø-Pores^	WCS	Transfer	Resistive	[46]
SnO_2_	PF	150 ^Ø-Pores^ 20 ^Ø-NPs^	WCS ^MA^	Transfer	Resistive	[45]
SnO_2_	NShs	6.3 ^CS^	WCS	Transfer	Resistive	[47]
Ce-doped SnO_2_	PF	10.3 ^CS-SnO2^	WCS	Transfer	Resistive	[73]
WO_3_	NNs	100 ^T^ 10,000 ^L^	CVD	Direct	Resistive	[48]
Au@WO_3_	NPs@NNs	100 ^T^ 10,000 ^L^ 3–10 ^S-NPs^	CVD	Direct	Resistive	[48]
Pt@WO_3_	NPs@NNs	100 ^T^ 10,000 ^L^ 1–5 ^S-NPs^	CVD	Direct	Resistive	[48]
Fe_2_O_3_@WO_3_	NPs@NNs	5–100 ^Ø-NNs^ 10,000 ^L-NNs^ 4–15 ^Ø-NPs^	CVD	Direct	Resistive	[49]
TiO_2_	NShs (NFls)	120 ^L^ 12–23 ^T^	WCS	Direct	Resistive	[51]
Pd@TiO_2_	NPs@NRds	20 ^Ø-NPs^ 100 ^Ø-NRs^ 4500 ^L-NRs^	CVD + WCS	Direct	Resistive	[52]
In_2_O_3_	NShs-hMSp (NFls)	20 ^T^	WCS	Transfer	Resistive	[50]
CuO	NShs (NFls)	2000 ^S^ 50 ^T^	WCS	Transfer	Resistive	[74]
Co_3_O_4_	cNCs	200 ^S^	WCS	Transfer	Resistive	[69]
Co_3_O_4_	ML	17.6 ^CS^	WCS	Transfer	Resistive	[72]
SiO_2_	NPs	-	-	Transfer	CTL	[53]
CeO_2_	NPs	-	-	Transfer	CTL	[53]
Y_2_O_3_	NPs	-	-	Transfer	CTL	[53]
La_2_O_3_	NPs	-	-	Transfer	CTL	[53]
Au@La_2_O_3_	NPs@NRds	-	WCS	Transfer	CTL	[119]
TiO_2_-SnO_2_	PF	-	WCS	Transfer	CTL	[102]
CuO/SnO_2_	PF	150 ^Ø−Pores^ 20 ^Ø-NPs^	WCS^MA^	Transfer	Resistive	[45]
ZnO-CuO	NShs	20–25 ^T^	PVD	Direct	Resistive	[75]
SiO_2_/Fe_3_O_4_	NPs	-	-	Transfer	CTL	[53]
ZnCo_2_O_4_	HSp	72 ^Ø^	WCS	Transfer	Resistive	[70]
Au	NPs	36 ^Ø^	WCS	Transfer	LSPR	[66]
Ag	NPs	51 ^Ø^	WCS	Transfer	LSPR	[67]

PF: porous film, NShs: nanosheets, NRgs: nanorings, NTPs: nanotetrapods, NFls: nanoflowers, NPs: nanoparticles, NRds: nanorods, NNs: nanoneedles, hMSp: hierarchical microsphere, cNCs: concave nanocubes, ML: monolayer, HSps: hollow spheres, PS: pore size, ^S^: size, ^T^: thickness, ^Ø^: diameter, ^OØ^: outer diameter, ^IØ^: inner diameter, ^L^: length, ^CS^: crystal size, WCS: wet chemical synthesis, PhA: photo-assisted, MA: microwave assisted, CVD: chemical vapor deposition, PVD: physical vapor deposition, QCM: quartz crystal microbalance, CTL: cataluminescence, LSPR: localized surface plasmon resonance.

**Table 9:** Summary of POMs and CbMs for VOCs sensing, their features and corresponding synthesis method and integration.

Material	Morphology	Features, nm	Method	Integration	Principle	Ref
Pt@MWCNTs	NPs/MWCNTs	2 ^Ø^/Up to 50,000 ^L^	CVD	Transfer	Resistive	[54]
Pd@MWCNTs	NPs/MWCNTs	3 ^Ø^/Up to 50,000 ^L^	CVD	Transfer	Resistive	[54]
PEDOT-PIL@Fe_3_O_4_	CSh (Ls^PEDOT^– MNPs^PIL@Fe3O4^)	15 ^S-NPs^	WCS	Transfer	Resistive	[116]
ZnO/MWCNTs	HSps^ZnO^-MWCNTs	300–350 ^Ø-HSps^ 30–40 ^T-HSps^	WCS	Transfer	Resistive	[106]
DT-capped Au	MLShs^DT^ NPs^Au^	100–300 ^FT^ 2–5 ^Ø- NPs^	WCS	Transfer	Resistive	[122]
MOF-ZnO	NCgs	100 ^S-NPs^ 60 ^CØ^ 25 ^PST^	WCS	Transfer	Resistive	[121]
SWCNTs/CdA	SWCNTs	1–5 ^Ø^ 1000–10,000 ^L^	WCS	Transfer	QCM	[123]
SWCNTs-calixarene	SWCNTs	-	WCS	Transfer	Resistive	[120]
PANI-CD	NPs	100	WCS	Transfer	Resistive	[68]
Ti-PTFE	Cls	10–30 ^NPs^	PVD	Transfer	Resistive	[109]
Ag-BC-bsh	NPs^Ag^ NPp	10 ± 7 ^Ø-NPs^ 45 ± 10 ^FØ^ >10,000 ^L-NPp^	WCS	Transfer	LSPR	[117]
Au-DDDT	NPs^Au^ Ls	4 ^Ø-NPs^/Up to 60 ^T-Ls^	LBL-SA	Transfer	Resistive	[124]
PEDOT-PSS/UL-GO	NShs^UL-GO^ PF	10,000–300,000 ^S-UL-GO^	PVD	Transfer	Resistive	[57]
Ag/SnO_2_/GO	NPs^Ag^	-	WCS	Transfer	Resistive	[110]
GO/ZnO	NRds^ZnO^ NShs^GO^	640 ^L-ZnO^	WCS	Transfer	Resistive	[111]
GO/ZnO/GO	NRds^ZnO^ NShs^GO^	640 ^L-ZnO^	WCS	Transfer	Resistive	[111]
MWCNTs/Au	MCWNTs-NPs^Au^	Up to 50,000 ^L-MWCNTs^ 3–15 ^OØ-MWCNTs^ 3–7 ^IØ-MWCNTs^ 2 ^Ø-Au^	CVD/WCS	Transfer	Resistive	[55]
MWCNTs/Au/MHDA	MCWNTs-NPs^Au^-ML^MHDA^	Up to 50,000 ^L-MWCNTs^ 3–15 ^OØ-MWCNTs^ 3–7 ^IØ-MWCNTs^ 2 ^Ø-Au^	CVD/WCS	Transfer	Resistive	[55]
NiO/MWCNTs	NPs/MWCNTs	25 ^Ø-NPs^ 20–35 ^Ø-MWCNT^	WCS	Transfer	Resistive	[105]
O_2_/MWCNTs	MWCNTs	50,000 ^L-MWCNTs^ 3–15 ^OØ-MWCNTs^ 3–7 ^IØ-MWCNTs^	CVD	Transfer	Resistive	[104]
FeO/MWCNTs	MWCNTs	50,000 ^L-MWCNTs^ 3–15 ^OØ-MWCNTs^ 3–7 ^IØ-MWCNTs^	CVD	Transfer	Resistive	[104]
PPy/rGo	NPs^−PPy^/NShs^−rGO^	80 ^Ø-NPs^	WCS	Transfer	Resistive	[58]
rGO/SnO_2_	NShs^−rGO^/NPs^−SnO2^	6–10 ^Ø-NPs^	WCS	Transfer	Resistive	[112]
Ag/PPy	NPs	17 ± 3 ^S-Au-NPs^ 44 ± 10 ^S-PPy-NPs^	WCS	Transfer	SAW	[107]
CdTe/PPy	QDs^−CdTe^/NPs^−PPy^	3.1 ± 0.7 ^S-QDs^ 35–55 ^S-NPs^	WCS	Transfer	SAW	[108]

MWCNTs: multi-walled carbon nanotubes, PEDOT: Poly(3,4-ethylenedioxythiophene), PIL: polymerized ionic liquid, DT: decanethiol, MOF: metal-organic frameworks, SWCNTs: single-walled carbon nanotubes, CdA: cadmium arachidate, PANI: polyaniline, CD: cyclodextrin, PTFE: polytetrafluoroethylene, Ag-BC: Ag nanoparticles embedded in bacterial cellulose nanopaper, bsh: blue shift, DDDT: 1,12-dodecanedithiol, PSS: poly(styrenesulfonate), UL-GO: ultra-large graphene oxide, GO: graphene oxide, MHDA: 16-mercaptohexadecanoic acid, PPy: polypyrrole, rGO: reduced graphene oxide, NPs: nanoparticles, CSh: core shell, Ls: layers MNPs: magnetic nanoparticles, HSps: hollow spheres, MLShs: monolayer shells, NCgs: hierarchical hollow nanocages, Cls: clusters, NPp: nanopaper, NShs: nanosheets, PF: porous film, NRds: nanorods, ML: monolayer, Ø: diameter, ^L^: length, ^S^: size, ^T^: thickness, ^FT^: film thickness, ^CØ^: cavity diameter, ^PST^: porous shell thickness, ^FØ^: fiber diameter, ^OØ^: outer diameter, ^IØ^: inner diameter, QDs: quantum dots, CVD: chemical vapor deposition, WCS: wet chemical synthesis, LBL-SA: layer-by-layer self-assembly, PVD: physical vapor deposition, QCM: quartz crystal microbalance, SAW: surface acoustic wave.

**Table 10:** A few literature examples of the application of e-noses in the area of air, water, and food quality, health care, and medicine. The table shows the number of sensors in the array, the targeted analytes, and data processing algorithms employed in each report.

Area	Objective	Sensor Elements	Number	Analytes	Algorithms	Ref.
Air quality and security	Indoor identification of low formaldehyde concentrations	Commercial MOX-Resistive	5	Formaldehyde	BP-ANN	[167]
	Identification of binary mixture: DMMP (ppb level) and methanol (sub ppm level)	Experimental MOX-Resistive MOX-SAW	4	DMMP, Methanol	PCA, ANN	[160]
	Detection of mixed indoor hazardous gases	Commercial MOX-Resistive	6	Methane, Formaldehyde, CO, Hydrogen	PCA, LDA, BP-ANN	[161]
Water quality	Discrimination polluted from clean water	Commercial MOX-Resistive	6	Propanol, Phenol, TFB, Benzene	PCA, HCA, SVM	[165]
	Identification of pollutants in water	Commercial MOX-Resistive	8	Ethanol, Acetone, Toluene, Ammonia, Ethylacetate	PCA, ANN, RBF-ANN, BP-ANN	[162]
	Identification of pollutants in water	Commercial MOX-Resistive	N/A	Chloroform Ammonia	PCA, LDA	[166]
Food quality	Off-flavors detection in alcoholic beverage (wine, beer)	N/A MOX-Resistive	18	Ethylacetate, TCA, 4-EP, Hexanol, Octenol, Diacetyl, BD	PCA, DFA	[168]
	Rice quality assessment—early detection of fungal infection	Commercial MOX-Resistive	12	Cotane, 2-pentylfuran, Dodecane, Toluene, Decane, Ethanol	PCA, LDA, SVM, PLS	[169]
	Fruit ripeness classification	Commercial MOX-Resistive *	7	Alkanes, Alcohols, Esters	PCA, LDA	[163]
Healthcare/medicine	Discrimination of pathogenic bacterial VOCs	Commercial MOX-Resistive Experimental OINCs-Resistive	6	Acetone, Formaldehyde, Ammonia, Ethanol, Ethylacetate, Acetic acid	PCA, CA	[170]
	Lung cancer and renal failure diagnosis	Experimental CbMs-Resistive	8	Acetone, Isoprene, Ammonia, Hydrothion	PCA, LDA	[164]
	Lung cancer diagnosis	Experimental MOX-Resistive	4	Formaldehyde	MVLR	[171]

* The classification of fruit ripeness is assisted by a digital camera system, OINCs: organic-inorganic nanocomposites, N/A: data not available, DMMP: dimethyl methylphosphonate, TFB: 1,3,5-trifluorobenzene, TCA: 2,4,6-trichloroanisole, EP: ethylphenol, BD: 2,3-butanedione, BP: back-propagation, ANN: artificial neural network, PCA: principal component analysis, LDA: linear discriminant analysis, HCA: hierarchical cluster analysis, SVM: support vector machine, RBF: radial basis function, DFA: discriminant factor analysis, PLS: partial least squares, CA: cluster analysis, MVLR: multivariate linear regression.

## Data Availability

Not applicable.
